# Management of patients with endometriosis and infertility: laparoscopic treatment and spontaneous pregnancy rate

**DOI:** 10.5935/1518-0557.20240018

**Published:** 2024

**Authors:** Yanina Rodríguez, Esteban Grasso, Lautaro Tessari, Florencia Perotti, Marcela Irigoyen, Antonio Cattaneo, A. Gustavo Martínez, Rosanna Ramhorst, Diego Gnocchi

**Affiliations:** 1 FERTILIS Medicina Reproductiva, Buenos Aires, Argentina; 2 Laboratorio de Inmunofarmacología, Instituto de Química Biológica de la Facultad de Ciencias Exactas y Naturales (IQUIBICEN) CONICET y Universidad de Buenos Aires, Argentina; 3 Facultad de Ciencias Exactas y Naturales, Universidad de Belgrano, Buenos Aires, Argentina; 4 Red Latinoamericana de Reproducción Asistida

**Keywords:** endometriosis, laparoscopic treatment, spontaneous pregnancy rate

## Abstract

**Objective:**

To evaluate factors involved in spontaneous pregnancy rate after surgery for endometriosis in patients with endometriosis and infertility.

**Methods:**

This retrospective study spanned from 2014 to 2020 and included a follow-up period of two years of patients with endometriosis-related infertility who underwent laparoscopic surgery. Women aged 25 to 43 years with patent tubes, no/mild male factor and no other infertility factors were selected and grouped according to fertility management as follows: patients immediately prescribed ART (16.5%, ART-p); patients who chose not to undergo ART (83.5%) and achieved spontaneous pregnancy (71.8% SP-p); and patients who first chose not to undergo ART but had it subsequently (28.2%, NSP-p).

**Results:**

A total of 200 patients were analyzed. Of the 167 patients who waited for spontaneous pregnancy, 71.8% achieved it. We observed a tendency of higher endometriosis ASRM scores in the ART-p group compared with patients who waited for spontaneous pregnancy, and lower scores in individuals that achieved spontaneous pregnancy. When we looked at how long it took to achieve pregnancy, we found that individuals in the SP-p group achieved pregnancy in 5.7 months, while subjects in the NSP-p group took 1.8 times longer than their peers in the SP-p group (*p*<0.001). However, once prescribed ART, the individuals in the NSP-p group achieved pregnancy within a similar time when compared with subjects in the SP-p group. In order to identify individuals that might benefit from ART early on, we performed a multivariable analysis and developed a decision tree (81.3% accuracy and 53.3% sensitivity).

**Conclusions:**

The present results indicated that, after surgery, the majority of patients achieved spontaneous pregnancy. The decision tree proposed in this study allows the early identification of patients who might require ART, thus decreasing the time between surgery and pregnancy and improving overall outcomes.

## INTRODUCTION

Endometriosis is currently seen as a systemic inflammatory disease associated with pelvic pain and infertility, among other symptoms (Chapron *et al.*, 2022; Pirtea *et al*., 2022). Though endometriosis is traditionally associated with pelvic manifestations, this disease displays multifactorial and systemic effects with a prevalence estimated between 2% and 10% in women in the general population (Vassilopoulou *et al*., 2018).

Endometriosis is also present in up to 50% of women with infertility (Vassilopoulou *et al*., 2018; Lee *et al*., 2020). Some of the mechanisms involved in endometrial pathogenesis may cause an exacerbated inflammatory state in the uterus and ovaries, thus affecting endometrial receptivity, ovarian reserve and oocyte quality (Chen *et al*., 2023). In the endometrium, the decidualization program is altered due to estradiol causing an increase in prostaglandin E2 production and resistance to progesterone, which affect the implantation rate (Zhang & Wang, 2023). Several other associations have been reported, such as aberrant gene expression in the endometrium associated with an increased production of inflammatory cytokines and chemokines, resulting in differential recruitment and differentiation of immune cells, reshaping immune response in the uterus and ovarian microenvironment (Vallvé-Juanico *et al*., 2019). All such factors contribute to subfertility via pelvic adhesions, distorted pelvic anatomy, and bilateral tubal blockage.

Therefore, early screening to select patients at higher risk of endometriosis is needed. The question is how to find these patients. Having patients answer a questionnaire is the first step in the diagnostic process (Chapron *et al*., 2022). Validated questionnaires for the early detection of patients at higher risk of endometriosis are currently available (Bailleul *et al*., 2021; Chapron *et al*., 2022). Apart from its effect on fertility, endometriosis is associated with dysmenorrhea, dyspareunia and lower abdominal pain; it may also cause dysuria and dyschezia, depending on the degree of involvement and location (Ekine *et al*., 2020). Taking all this into account, it is important to consider the patient’s clinical symptoms, perform adequate physical examination, and order complementary tests including imaging-based approaches, such as ultrasound or magnetic resonance imaging (MRI), to diagnose ovarian and deep infiltrating endometriosis (Chapron *et al*., 2022). Unfortunately, imaging-based approaches are poor at diagnosing superficial endometriosis, which may require diagnostic laparoscopy (Goncalves *et al*., 2021).

Until the last decade, diagnostic laparoscopy was routinely performed for the diagnosis and treatment of endometriosis in patients with suspected endometriosis who consulted for pain and infertility. More recently, however, diagnostic laparoscopy has been less prescribed and performed (Pirtea *et al*., 2022). This is due to the accumulated evidence suggesting that surgery for endometriosis does not necessarily improve assisted reproductive technology (ART) treatment outcomes (Pirtea *et al.*, 2022). In fact, reports have indicated that surgery may cause further harm by impairing the ovarian reserve (Benaglia *et al.*, 2017). Contrary to observations made in ovarian stimulation, ART does not worsen endometriosis symptoms and has no impact on ovarian endometriomas or deep infiltrating endometriosis (Somigliana *et al*., 2019).

Given this controversy, it is possible that only a subgroup of patients with endometriosis-associated infertility might benefit from laparoscopic treatment and improve their chances of conceiving naturally. In this regard, Vercellini *et al*. (2009) reported evidence indicating that surgery for pelvic endometriosis increased the chances of conceiving naturally by approximately 50% in the 12-18 months after surgery. This was also confirmed by others authors (Dückelmann *et al*., 2021; Muzii *et al.*, 2021). Thus, while seeing patients with clinical suspicion of endometriosis and infertility, we must consider their age, ovarian reserve, tubal patency and male factor among other clinical parameters, to thus evaluate their chances of conceiving naturally as wells as the potential benefit of surgical treatment (Rizk *et al*., 2015; Lee *et al*., 2020; Dückelmann *et al*., 2021; Khan & Lee, 2021; Muzii *et al*., 2021).

Endometriosis-associated infertility is still being debated and more studies are required, especially considering that the high efficacy of modern-day assisted reproductive technology (ART) has led to progressively adopting ART-first approaches, particularly for women with endometriosis (Pirtea *et al.*, 2022). However, surgery is still recommended for some patients with endometriosis depending on the symptoms they present with and whether they wish to become pregnant. The following questions must be answered: Does laparoscopy play a role in these patients? What other factors are involved in the achievement of spontaneous pregnancy by patients with endometriosis? This study evaluated the spontaneous pregnancy rate of patients with endometriosis after surgical treatment and its possible associations with different clinical factors.

## MATERIALS AND METHODS

This retrospective observational study used the anonymized records of patients with endometriosis-related infertility who underwent laparoscopic surgery at “Fertilis - Sanatorio Las Lomas”, from 2014 to 2020 with up to two years of follow up. All patients included had indication for endometrial surgery due to their symptoms.

Of the 303 patients that met the inclusion criteria (age 25-43 years; symptoms and/or image findings consistent with endometriosis; infertility; and laparoscopic diagnosis of endometriosis), 200 also met the exclusion criteria (other laparoscopic diagnosis without findings compatible with endometriosis; history of previous surgeries for endometriosis; bilateral negative tubal patency; thrombophilia; recurrent abortion; and moderate/severe male factor) and were used in statistical analysis.

The following data were collected from patient medical charts: fertility treatment, age (grouped as <30, 30-34, 35-39 and >39 years), endometriosis, ASRM score, initial symptoms, primary/secondary infertility, time of infertility, tubal and uterine quality and time until pregnancy. The rASRM classification was designed to categorize cases of endometriosis via direct visualization of the pelvic organs during laparoscopy or laparotomy into four stages: minimal (I), mild (II), moderate (III), and severe (IV). Changes involving the peritoneum, the fallopian tubes and ovaries are used to stage the disease. When using the rASRM system, different points are assigned depending on whether the endometriotic lesion is deep or superficial, the size of the endometriotic lesion, and the type (filmy or dense) and extent of adhesions involving the fallopian tubes, ovaries, and the pouch of Douglas. The points are added to a total score, and the total score is used to stratify the disease into one of the four stages (Hudelist *et al*., 2021)ureters, bowel and sacral roots. Adenomyosis (growth of endometrium in the myometrium, sometimes explained by disruption of the uterine junctional zone. Table 1 shows some of the collected information. Patients were initially categorized according to fertility treatment as patients that used ART immediately after surgery (16.5%, ART-p) and patients that waited for a spontaneous pregnancy (83.5%). This last group was subdivided into patients that achieved spontaneous pregnancy within 12 months of surgery (71.8%, SP-p) and individuals unable to achieve spontaneous pregnancy who required ART (28.2%, NSP-p).

**Table 1:** Demographic information of the studied patients.

Age (mean±SD)	35.2±3.4 (range 25-43) years
Infertility time (mean±SD)	23.4±13.6 (range 6-96) months
Dysmenorrhea	88.5% (177)
No previous ART	81.5% (163)
Endometriosis ASRM score	EAS I: 15.5% (31)
	EAS II: 38% (76)
	EAS III: 38.5% (77)
	EAS IV: 8% (16)
Tubal quality	Regular: 14.5% (29)
	Good:. 85.5% (171)
EFI score	7.05±0.09 (range 3-10)

Data was analyzed with GraphPad Prism 9.4 (GraphPad Software) using chi-square, ANOVA and T-test depending on each comparison. The decision tree was made using RPart-package on R (R Core Team, 2022; Therneau & Atkinson, 2023). Different values for the decision tree parameters, such as maximum depth and minimal records per node, were tested to optimize accuracy and sensitivity.

## RESULTS

**Figure 1:**
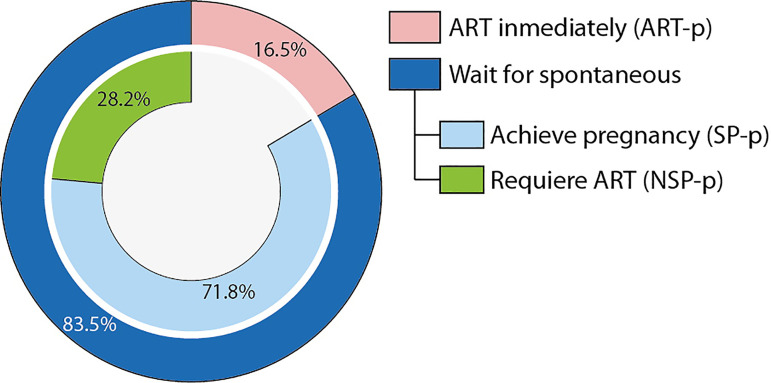
Distribution of patients according to initial fertility treatment and overall outcome.

**Figure 2:**
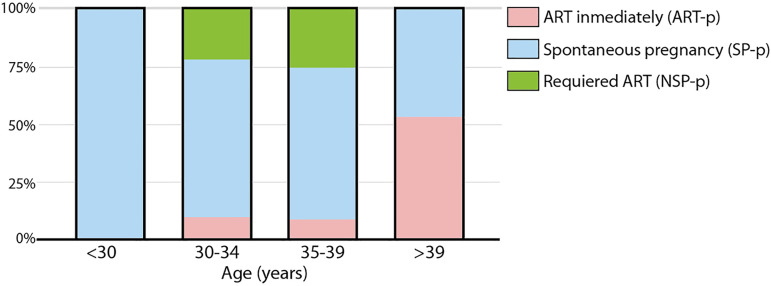
Patient age according fertility treatment. ART-p are overrepresented in the 39+ years group, which could be caused by other factors associated to age such as low ovarian reserve.

**Figure 3:**
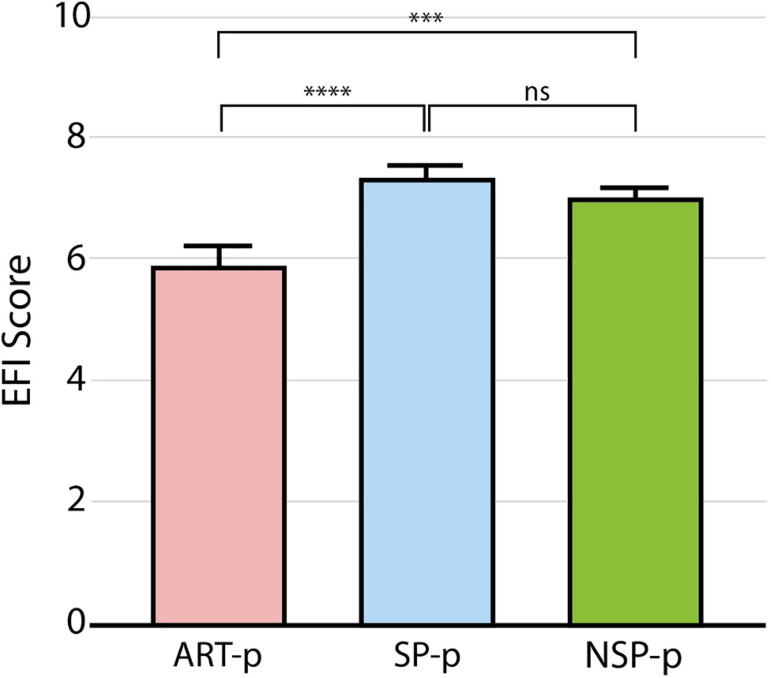
EFI score. Patients prescribed ART immediately after surgery (ART-p) presented a significantly lower EFI score in comparison with both groups that opted for spontaneous pregnancy (SP-p, NSP-p). Interestingly, no significant difference was found between these last two groups. Mean±SEM; Anova, sidak post test ***p<0.001, ****p<0.0001.

From the initial 200 patients, 16.5% opted for immediate ART after laparoscopic treatment, while the rest opted to wait for spontaneous pregnancy, of which 71.8% were able to achieve it within 12 months (Figure 1). As we further evaluated the treatment approaches within each age group, we found that the patients who chose to undergo ART immediately after surgery were overrepresented in the older group (>39 years). This was expected as other factors associated with older age and unrelated to endometriosis might have been involved in the medical decision to go for ART immediately after laparoscopic treatment (Figure 2). Furthermore, when we calculated the EFI score, we found that patients with ART as the initial conduct had a lower score than those who opted to wait for spontaneous pregnancy, which supports the idea that other factors might be involved. Interestingly, we did not observe differences between the patients that achieved spontaneous pregnancy (SP-p) and the ones that did not (NSP-p) (Figure 3).

**Figure 4:**
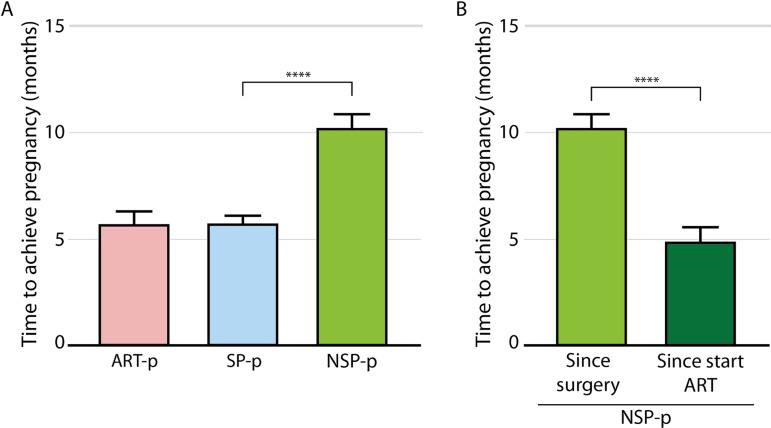
Time to achieve pregnancy. (A) Patients in the NSP group took significantly longer to achieve pregnancy since surgery than subjects in the SP group. However, (B) this difference disappears if only the time since the change in treatment is considered. Mean±SEM; Anova, sidak post test ****p<0.0001.

Then, we focused on the patients that opted to wait for a spontaneous pregnancy. When we evaluated how long it took to achieve pregnancy, we found that the individuals in the SP-p group achieved pregnancy in 5.7 months, while the subjects on the NSP-p group took almost 1.8 times longer (10.2±3.7 *vs*. 5.7±3.6 months, *p*<0.0001) (Figure 4A). Interestingly, when only the time since the treatment change from waiting for spontaneous pregnancy to ART was considered, we found that patients achieved pregnancy within a similar time than the ones in the SP group (4.9±3.7 months), suggesting that patients in the NSP group might have benefited if they had been identified earlier (Figure 4B).

**Figure 5:**
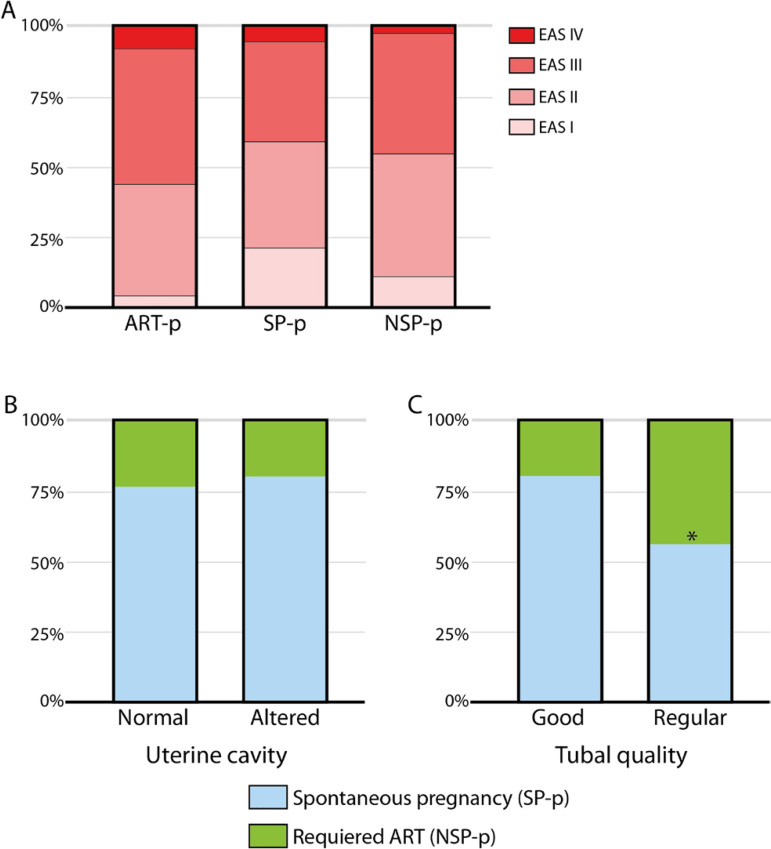
A- ASRM score by patient group. Patients in the ART-p group showed a tendency toward higher ASRM scores (III and IV), while individuals in the SP-p group showed a tendency toward lower ASRM scores (I and II). No significant difference was found. B- Comparison of uterine cavity quality between patients in the NSP and SP groups. No differences were found. C- Comparison of tubal quality between patients in the NSP and SP groups. NSP had lower tubal quality. Chi-square test.

With this in mind, we decided to look for differences in the other recorded parameters between the SP-p and NSP-p groups that might be useful to predict the outcome of patients who chose to wait. We did not find significant differences in the ASRM score, though a higher proportion of patients with lower ASRM scores (I) were in the SP (21.2%) *vs*. the NSP (11.4%) group. Interestingly, we found that individuals in the ART-p group tended to have higher ASRM scores (III and IV) than the patients who chose to wait for a spontaneous pregnancy (Figure 5A). Of all other studied variables, only tubal quality showed a significant difference, with a higher percentage of regular quality on the NSP-p group (22.8% *vs*. 8.5%, *p*<0.05) (Figures 5B, 5C).

**Figure 6:**
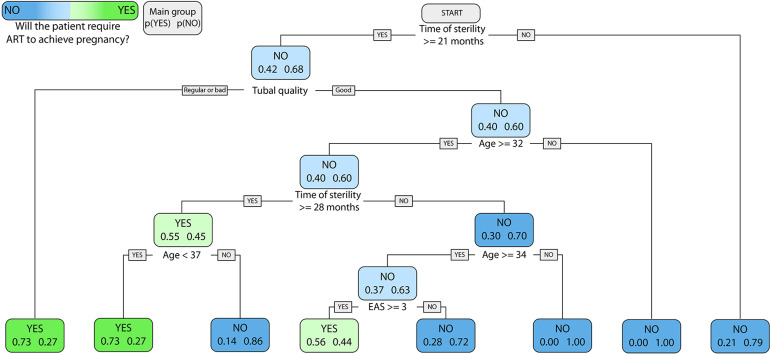
Decision tree. A multivariable approach was used to predict the patients that required ART after waiting for spontaneous pregnancy. Figure shows the optimized decision tree, accuracy 81.3%, sensitivity 53.3%.

Since none of the studied variables alone was able to identify patients in need of ART, we performed a multivariate analysis. As age could be associated with other factors and considering that in our study all patients in the NSP group were aged between 30 and 39 years, we chose to focus on patients younger than 40 years old, since they might be the ones that benefit the most from ART. Using R, we obtained a decision tree with 81.3% accuracy and 53.3% sensitivity on the original data set (Figure 6).

## DISCUSSION

As indicated in previous clinical studies about the management options for endometriosis-related infertility, our data also pointed to an increase in spontaneous pregnancy after surgical treatment (Dückelmann *et al*., 2021; Muzii *et al*., 2021). This suggests that there is a group of patients (normal ovarian reserve, normal patency and mild male factor) who might benefit from laparoscopic infertility treatment associated with endometriosis to improve their chances of conceiving naturally (Rizk *et al.*, 2015; Muzii *et al.*, 2021).

Chronic inflammation can impair ovarian or endometrial function, leading to disorders of folliculogenesis or implantation (Benaglia *et al*., 2017; Pirtea *et al.*, 2022). Endometriosis usually develops with diminished ovarian reserve due to the presence of an inflammatory microenvironment. The identification of progesterone resistance in an eutopic endometrium leads to an estrogenic state that affects endometrial receptivity (Lessey *et al*., 1996; Zeitoun & Bulun, 1999; Kao *et al*., 2003; Burney *et al*., 2007; Lessey & Kim, 2017). Although we did not find a significant correlation between ASRM score and spontaneous pregnancy, a tendency toward lower scores in association with better outcomes was identified. A higher proportion of high ASRM scores was observed among the patients who chose to wait for spontaneous pregnancy instead of undergoing ART immediately, possibly indicating the presence of other associated factors not considered in this study.

Although the majority of the patients who opted to wait for spontaneous pregnancy after endometrial surgery achieved it within 12 months, we found a group of individuals that was not able to get pregnant spontaneously and eventually required ART. Consequently, these patients had a longer time from surgery to pregnancy. Interestingly, when we looked at how long these patients took to achieve pregnancy since the start of ART, we found that they took a similar amount of time than those who achieved it spontaneously. If identified earlier, the patients who required ART might have achieved pregnancy by five to ten months earlier. This is not only relevant from the psychological point of view (Assaysh-Öberg *et al*., 2023; Tetecher *et al*., 2024), but also from an endometrial perspective, since laparoscopic surgery for endometriosis is not curative, with 40-45% of women having recurring disease, which may, again, interfere with fertility (Vercellini *et al*., 2009).

In order to identify the patients that will require ART at the clinic, we studied several clinical parameters, including age, ASRM score, tubal and uterine cavity quality, among others. Although we found an association between some of these parameters and patient outcomes, none was able to identify patients in need of ART. As a result, we performed a multivariate analysis. Considering that the goal was to identify patients at the clinic, we chose to develop a decision tree algorithm. This type of algorithm presents several advantages. It is not only very easy to use at the clinic, but it also provides for machine learning opportunities, it is statistically driven, flexible and can find patterns hidden in the data (Therneau & Atkinson, 2023). One of the main points about the flexibility of such type of algorithm is that it considers that the same clinical parameter might lead to different outcomes depending on other parameters. For example, patient age may lead to different predictions depending on how long the patient has been infertile for.

We generated a decision tree with 81.3% accuracy and 53.3% sensitivity from the original set of data. Our decision tree requires only four parameters (time of infertility, tubal quality, age and ASRM score) and can be worked through in less than a minute without other tools or calculations, which makes it ideal for implementation at the clinic and a tool that might result in shorter waiting times until pregnancy for a significant part of the patients with endometriosis-related infertility. Further studies with more patients and variables might further improve the proposed decision tree.

Another tool to evaluate which is the best approach for patients with endometriosis is the Endometrial Fertility Index, or EFI (Adamson & Pasta, 2010). The EFI score system has been developed using a wide variety of endometrial patients and validated several times, proving to be especially useful for patients with poor prognosis (Adamson & Pasta, 2010; Adamson, 2013). In contrast, the decision tree algorithm developed herein focuses on patients that have a good EFI score and aims to complement the EFI by helping to identify those patients that, even with a good EFI score, will probably require ART. The average EFI score of the patients used in the model was 7.27, ranging from 4 to 10.

The management of endometriosis-associated infertility is still a topic of discussion, especially in what concerns the role of surgery (Rizk *et al.*, 2015; Lee *et al*., 2020; Bailleul *et al*., 2021; Muzii *et al*., 2021). The results presented herein support other studies that suggested that surgical treatment for endometriosis might improve spontaneous pregnancy rates. Furthermore, we propose that the early identification of patients in need of ART to achieve pregnancy after the surgery will decrease the time between surgery and pregnancy and thus improve overall outcomes.

## CONCLUSION

The decision tree obtained in the present study might be a useful tool to identify patients with good EFI scores who might need ART after endometrial surgery.

## References

[r1] Adamson GD. (2013). Endometriosis Fertility Index: is it better than the present
staging systems?. Curr Opin Obstet Gynecol.

[r2] Adamson GD, Pasta DJ. (2010). Endometriosis fertility index: the new, validated endometriosis
staging system. Fertil Steril.

[r3] Assaysh-Öberg S, Borneskog C, Ternström E. (2023). Women’s experience of infertility & treatment - A silent
grief and failed care and support. Sex Reprod Healthc.

[r4] Bailleul A, Niro J, Du Cheyron J, Panel P, Fauconnier A. (2021). Infertility management according to the Endometriosis Fertility
Index in patients operated for endometriosis: What is the optimal time
frame?. PLoS One.

[r5] Benaglia L, Castiglioni M, Paffoni A, Sarais V, Vercellini P, Somigliana E. (2017). Is endometrioma-associated damage to ovarian reserve progressive?
Insights from IVF cycles. Eur J Obstet Gynecol Reprod Biol.

[r6] Burney RO, Talbi S, Hamilton AE, Vo KC, Nyegaard M, Nezhat CR, Lessey BA, Giudice LC. (2007). Gene expression analysis of endometrium reveals progesterone
resistance and candidate susceptibility genes in women with
endometriosis. Endocrinology.

[r7] Chapron C, Lafay-Pillet MC, Santulli P, Bourdon M, Maignien C, Gaudet-Chardonnet A, Maitrot-Mantelet L, Borghese B, Marcellin L. (2022). A new validated screening method for endometriosis diagnosis
based on patient questionnaires. EClinicalMedicine.

[r8] Chen LH, Lo WC, Huang HY, Wu HM. (2023). A Lifelong Impact on Endometriosis: Pathophysiology and
Pharmacological Treatment. Int J Mol Sci.

[r9] Dückelmann AM, Taube E, Abesadze E, Chiantera V, Sehouli J, Mechsner S. (2021). When and how should peritoneal endometriosis be operated on in
order to improve fertility rates and symptoms? The experience and outcomes
of nearly 100 cases. Arch Gynecol Obstet.

[r10] Ekine AA, Fülöp I, Tekse I, Rúcz Á, Jeges S, Koppán Á, Koppán M. (2020). The Surgical Benefit of Hysterolaparoscopy in
Endometriosis-Related Infertility: A Single Centre Retrospective Study with
a Minimum 2-Year Follow-Up. J Clin Med.

[r11] Goncalves MO, Siufi Neto J, Andres MP, Siufi D, de Mattos LA, Abrao MS. (2021). Systematic evaluation of endometriosis by transvaginal ultrasound
can accurately replace diagnostic laparoscopy, mainly for deep and ovarian
endometriosis. Hum Reprod.

[r12] Hudelist G, Valentin L, Saridogan E, Condous G, Malzoni M, Roman H, Jurkovic D, Keckstein J. (2021). What to choose and why to use - a critical review on the clinical
relevance of rASRM, EFI and Enzian classifications of
endometriosis. Facts Views Vis Obgyn.

[r13] Kao LC, Germeyer A, Tulac S, Lobo S, Yang JP, Taylor RN, Osteen K, Lessey BA, Giudice LC. (2003). Expression profiling of endometrium from women with endometriosis
reveals candidate genes for disease-based implantation failure and
infertility. Endocrinology.

[r14] Khan S, Lee CL. (2021). Treating Deep Endometriosis in Infertile Patients before Assisted
Reproductive Technology. Gynecol Minim Invasive Ther.

[r15] Lee D, Kim SK, Lee JR, Jee BC. (2020). Management of endometriosis-related infertility: Considerations
and treatment options. Clin Exp Reprod Med.

[r16] Lessey BA, Kim JJ. (2017). Endometrial receptivity in the eutopic endometrium of women with
endometriosis: it is affected, and let me show you why. Fertil Steril.

[r17] Lessey BA, Yeh I, Castelbaum AJ, Fritz MA, Ilesanmi AO, Korzeniowski P, Sun J, Chwalisz K. (1996). Endometrial progesterone receptors and markers of uterine
receptivity in the window of implantation. Fertil Steril.

[r18] Muzii L, DI Tucci C, Galati G, Mattei G, Chinè A, Cascialli G, Palaia I, Benedetti Panici P. (2021). Endometriosis-associated infertility: surgery or
IVF?. Minerva Obstet Gynecol.

[r19] Pirtea P, Vulliemoz N, de Ziegler D, Ayoubi JM. (2022). Infertility workup: identifying endometriosis. Fertil Steril.

[r20] R Core Team (2022). R: A language and environment for statistical computing.

[r21] Rizk B, Turki R, Lotfy H, Ranganathan S, Zahed H, Freeman AR, Shilbayeh Z, Sassy M, Shalaby M, Malik R. (2015). Surgery for endometriosis-associated infertility: do we
exaggerate the magnitude of effect?. Facts Views Vis Obgyn.

[r22] Somigliana E, Viganò P, Benaglia L, Busnelli A, Paffoni A, Vercellini P. (2019). Ovarian stimulation and endometriosis progression or recurrence:
a systematic review. Reprod Biomed Online.

[r23] Tetecher L, Cocchiaro T, Guarino A, Giannini T, Maucione S, Di Trani M, Rago R, Ciocca G. (2024). Sexological and traumatic aspects in reproductive difficulties: a
psychometric study on couples seeking help for infertility. J Endocrinol Invest.

[r24] Therneau T, Atkinson B. (2023). rpart: Recursive Partitioning and Regression Trees.

[r25] Vallvé-Juanico J, Houshdaran S, Giudice LC. (2019). The endometrial immune environment of women with
endometriosis. Hum Reprod Update.

[r26] Vassilopoulou L, Matalliotakis M, Zervou MI, Matalliotaki C, Spandidos DA, Matalliotakis I, Goulielmos GN. (2018). Endometriosis and in vitro fertilisation. Exp Ther Med.

[r27] Vercellini P, Barbara G, Abbiati A, Somigliana E, Viganò P, Fedele L. (2009). Repetitive surgery for recurrent symptomatic endometriosis: what
to do?. Eur J Obstet Gynecol Reprod Biol.

[r28] Zeitoun KM, Bulun SE. (1999). Aromatase: a key molecule in the pathophysiology of endometriosis
and a therapeutic target. Fertil Steril.

[r29] Zhang P, Wang G. (2023). Progesterone Resistance in Endometriosis: Current Evidence and
Putative Mechanisms. Int J Mol Sci.

